# Assessment of pulse rate variability by the method of pulse frequency demodulation

**DOI:** 10.1186/1475-925X-4-62

**Published:** 2005-11-01

**Authors:** Junichiro Hayano, Allan Kardec Barros, Atsunori Kamiya, Nobuyuki Ohte, Fumihiko Yasuma

**Affiliations:** 1 Core Laboratory, Nagoya City University Graduate School of Medical Sciences, Nagoya 467-8601, Japan; 2 Universidade Federal do Maranhao, Sao Luis-Ma, Brazil; 3 Department of Cardiovascular Dynamics, National Cardiovascular Center Research Institute, Suita, Osaka 565-8565, Japan; 4 Department of Internal Medicine and Pathophysiology, Nagoya City University Graduate School of Medical Sciences, Nagoya 467-8601, Japan; 5 Department of Internal Medicine, Suzuka National Hospital, Suzuka 513-8501, Japan

## Abstract

**Background:**

Due to its easy applicability, pulse wave has been proposed as a surrogate of electrocardiogram (ECG) for the analysis of heart rate variability (HRV). However, its smoother waveform precludes accurate measurement of pulse-to-pulse interval by fiducial-point algorithms. Here we report a pulse frequency demodulation (PFDM) technique as a method for extracting instantaneous pulse rate function directly from pulse wave signal and its usefulness for assessing pulse rate variability (PRV).

**Methods:**

Simulated pulse wave signals with known pulse interval functions and actual pulse wave signals obtained from 30 subjects with a trans-dermal pulse wave device were analyzed by PFDM. The results were compared with heart rate and HRV assessed from simultaneously recorded ECG.

**Results:**

Analysis of simulated data revealed that the PFDM faithfully demodulates source interval function with preserving the frequency characteristics of the function, even when the intervals fluctuate rapidly over a wide range and when the signals include fluctuations in pulse height and baseline. Analysis of actual data revealed that individual means of low and high frequency components of PRV showed good agreement with those of HRV (intraclass correlation coefficient, 0.997 and 0.981, respectively).

**Conclusion:**

The PFDM of pulse wave signal provides a reliable assessment of PRV. Given the popularity of pulse wave equipments, PFDM may open new ways to the studies of long-term assessment of cardiovascular variability and dynamics.

## Background

Analysis of heart rate variability (HRV) has been standardized with using R-R intervals of electrocardiogram (ECG) as the source signal [1]. The measurement of ECG, however, requires multiple electrode attachments and cable connections, which precludes frequent assessments of HRV in general populations. Beyond the uses as an autonomic functional index [2,3] or as a prognostic marker of long-term survival [4-6], the applications of HRV have extended into many areas of health sciences and technologies, such as those as markers for assessing the levels of physical and mental demands [7,8], the degree of fatigue [9], the depth of relaxation and resting [10,11] and the comfortableness of living and occupational environments [12,13]. For these applications, self-applicable devices that allow frequent, preferably everyday measurement in general population may be more useful.

From this aspect, analysis of pulse rate variability (PRV) from pulse wave signal has been studied as a potential surrogate of HRV analysis [14]. In contrast to ECG, pulse wave can be recorded with a single sensor without electrode and indeed, pulse wave equipments are popular and widely used not only in hospital cares but also in health sciences and clinical homecare practices. However, there is also a problem, i.e., smoother waveform of pulse wave precludes accurate measurement of pulse-to-pulse intervals by fiducial-point algorithms such as those used for measuring R-R intervals of ECG.

Here, we report a technique of pulse frequency demodulation (PFDM) as a method for estimating the function of instantaneous pulse rate. Taking advantage of smoother waveform of pulse wave, the PFDM extracts instantaneous pulse rate directly from pulse wave signal as a function of time. The continuous function of pulse rate can be converted into instantaneous pulse interval function, which can be directly used for spectral analysis. In this study, we tested the performance of the PFDM using both simulated data and actual pulse wave signals that were recorded with a wireless trans-dermal photoelectric device. We also compared PRV estimated by the PFDM with HRV measured by R-R intervals of simultaneously recorded ECG.

## Methods

### Principle of PFDM

The core process of PFDM is frequency demodulation based on the method of complex demodulation (CDM) [15-17]. CDM is a non-linear time-domain method for time series analysis, which provides amplitude and frequency of non-stationary/unstable oscillatory signal as a continuous function of time. Principle and computer algorithm of CDM have been published previously [16]. Briefly, CDM extracts the time dependent functions of instantaneous frequency through the following four steps: (1) the spectral region of interest (the frequency range of target oscillation) is shifted to zero frequency by forming a product, throughout the record, of the original signal and a complex sinusoid at a reference frequency (Fr, the center frequency of the spectral region of interest), (2) the resultant complex signal is low-pass filtered so that only frequency components around zero remain, (3) the real and imaginary parts of the low-pass filtered signal are converted to a polar form, yielding the instantaneous phase, as a function of time, of the component identified at or near the Fr, and (4) the time series of frequency of the target oscillation is calculated through adding the first-order derivative of the phase to the Fr, since the slope of the phase versus time curve indicates deviation of instantaneous operative frequency from the Fr.

In PFDM, the CDM is customized for analyzing pulse wave signal. Pulse frequency (instantaneous pulse rate) could change widely from less than 0.5 Hz (30 bpm) to more than 3 Hz (180 bpm). Because CDM extracts frequency of oscillatory components within an assigned spectral region (range of CDM filter), an appropriate frequency range needs to be selected so that it covers the possible range of pulse frequency. This is, however, a trade-off with the requirement that the frequency range of CDM filter needs to be narrow enough to avoid influence of harmonics and subharmonics of the fundamental oscillation (pulse wave).

When frequency of pulse wave decreases to a frequency as low as *f *Hz, the upper limit of the frequency range should be less than 2*f *Hz (frequency of the 2nd harmonic of the pulse wave). Likewise, when frequency of pulse wave increases to a frequency as high as *f *Hz, the lower limit of the frequency range should be greater than *f*/2 Hz (frequency of the 2nd subharmonic of the pulse wave). If the range for CDM filter is expressed as Fr ± Fc, where Fr is the reference frequency (the center frequency of the spectral region of interest) and Fc is the corner frequency of the low-pass filter, then the necessary condition for avoiding the influence of harmonics and subharmonics is

Fc < Fr/3.

For example, when mean pulse rate is 60 bpm, the widest possible range for CDM filter is 60 ± 20 (40 to 80) bpm; if pulse frequency deviates from this range during analyzing period, CDM would not provide an adequate estimation of pulse rate.

To overcome this problem, we devised the algorithm of PFDM incorporating the following three features: (1) overlapping short-segmentation of data, (2) adaptive determination of the Fr, (3) iterative algorithm for optimizing Fr, and (4) stepwise convergence of Fc toward Fr/3 during the iterative process. Briefly, data are first divided into short segments so that pulse frequency within each segment can be expected to remain within the range that can be covered by a feasible CDM filter. In each segment, the mean pulse frequency of the previous segment is used as the initial Fr value (adaptation). The Fr is further optimized through iteration processes, i.e., mean pulse frequency calculated is iteratively used as the Fr in the next iteration until the mean pulse frequency calculated agrees with the Fr used. Finally, to further guard against the possibility that pulse frequency deviates from the range of CDM filter, an Fc of Fr/2 is used for the initial process and it is converged to Fr/3 in the following iteration processes. The effectiveness of each of these processes is demonstrated in the simulation studies.

### Simulation data

**Figure 1:**
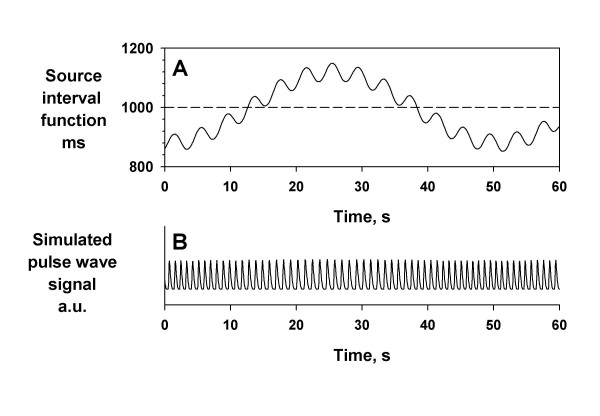
**Method for generating simulated pulse wave signal. **Simulated pulse wave (panel B) was generated as a sequence of a unit pulse wave. The pulse interval was modulated by source interval functions (panel A). The width of each unit pulse wave was also modulated as 18.97 × (PI)^1/2 ^ms, where PI is the pulse interval. a.u. = arbitrary unit.

Simulated pulse wave signal was generated as a sequence of a unit pulse wave that was sampled from actual pulse wave of a healthy subject. The pulse interval was modulated by various source interval functions (modulator functions), which were used as the reference signals for evaluating the performance of PFDM (Fig. 1). The width of each unit pulse wave was also modulated according to the changes in pulse interval as 18.97 × (PI)^1/2 ^ms, where PI is the pulse interval.

### Measurement of actual data

**Figure 2:**
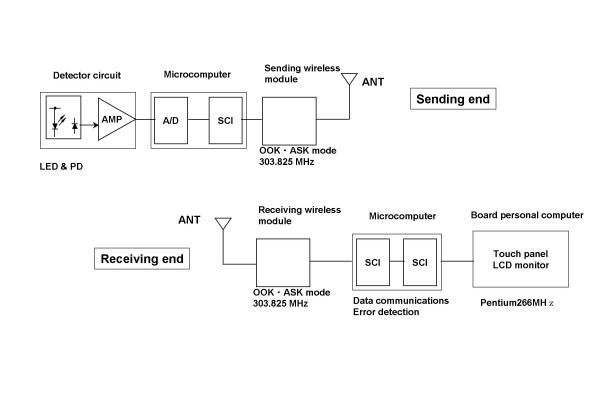
**Block diagram of wireless trans-dermal photoelectric pulse wave monitoring system. **This system detects pulse wave as the amount of light absorption by erythrocyte hemoglobin in pulsating epidermal capillary bed. Green light is emitted from a light-emitting diode (LED) to the skin and the reflected light is detected by a photodiode (PD) embedded in a sensor tip. The detected signal is amplified, digitized by an analog-to-digital (A/D) converter and processed with a serial signal conversion interface (SCI). The signal is then transmitted from a wireless micro-power module (303.825MHz). The signal is received with a receiving wireless module and processed by a microcomputer for data communications and error detection. The pulse wave signals are sent to board PC on which time series analyses including PFDM are performed and the results are presented on a liquid crystal display (LCD). AMP = amplifier, ANT = antenna, ASK = amplitude shift keying, OOK = on-off keying.

**Figure 3:**
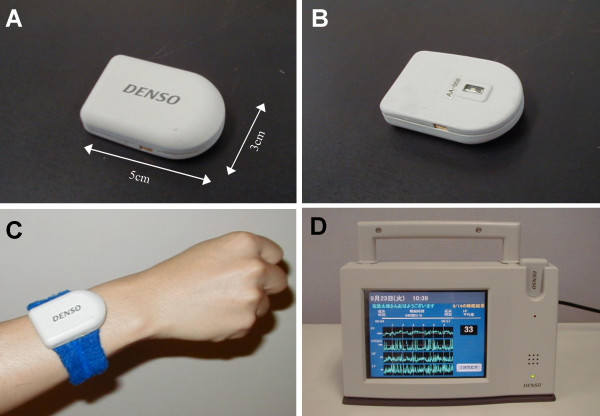
**A prototype of the wireless trans-dermal photoelectric pulse wave monitoring system (DENSO, Japan). **Panels A, B and C show a pulse wave sensor unit that applies green light to the skin and detects the reflected light through a small window (panel B) on the reverse side of the unit facing to the skin. The sensor unit transmits pulse wave signal to a receiver unit (panel D). The receiver unit has a small personal computer with LCD. The unit also has a slot used for charging the battery of the sensor unit.

We studied 33 subjects (23 males and 10 females); the mean age (standard deviation [SD]) was 34 (7) yr and age range was 22–47 yr. They were recruited from the staffs in the work places of the authors. All subjects gave their written informed consent and the procedure was performed according to the Ethical Guidelines of Nagoya City University Graduate School of Medical Sciences. Continuous pulse wave was recorded from the dorsal side of the wrist with a wireless trans-dermal photoelectric pulse wave sensor system (Prototype C, DENSO, Japan, Fig. 2 and 3), by which the pulse wave data were digitized (14 bit). ECG was recorded simultaneously with a digital Holter recorder (RAC-2102, Nihon Koden, Tokyo, Japan). In order to obtain exact matching of time between the two recordings, several event markers were also recorded simultaneously. Both continuous pulse wave and ECG data were recorded during all-night sleep in a laboratory chamber, because the pulse wave device allowed stable measure of pulse wave signal only during rest at present. To our knowledge, no noninvasive ambulatory devices are currently available for measuring stable pulse wave signal during activities.

### Data analysis

For both simulated and actual data analysis, pulse wave data were sampled at 20 Hz. The PFDM was performed with custom-made software written with FORTRAN 95 (Salford Software Ltd, Old Trafford, Manchester, UK). For the PFDM, the data segments had a length of 30 s with overlapping for 10 s at both ends. For the iteration for optimizing Fr, the tolerance for the difference between the mean pulse frequency and Fr was set at 0.001 bpm. Although the instantaneous pulse frequencies were calculated for every sampling point (at 20 Hz), they were averaged over every 500 ms and converted into pulse intervals in order to compare with the source interval functions (for the simulation data) or R-R intervals of ECG (for the actual data).

HRV, by definition, is the beat-to-beat variability of sinus rhythm [1,18]. Thus, all R-R interval data involving ectopic beat(s) or heart block(s) were excluded from the analysis. The pulse wave data, however, have less information about arrhythmias. An ectopic beat, either supra-ventricular or ventricular, could result in consecutive short pulse intervals, a long pulse interval or even a normal interval depending on the timing and whether it generates a detectable pulse wave or not. Heart blocks may also result in long intervals. To avoid the effects of rhythm disturbances and noises on the analysis of PRV, we excluded all *abnormal *pulse intervals, which were defined as those deviating 12% or more from the local moving average over the preceding 20 s. The definition of abnormal pulse interval was determined tentatively considering expected range of physiological PRV for the subject population.

The ECG data were analyzed with a Holter scanner (DSC-3100, Nihon Koden, Tokyo, Japan), on which the results of automatic labeling of QRS complexes were reviewed and manually edited for all errors. The ECG were analyzed with a sampling frequency of 125 Hz and, thus, the R-R intervals were measured at a time resolution of 8 ms. The time series data of R-R interval were interpolated along the time axis with a horizontal-step function (R-R interval was considered as constant during each R-R interval) and resampled at 2 Hz.

### Spectral analysis

Fast Fourier transformation with a Hanning window was performed for sequential 5-minute segments of both pulse interval and R-R interval data. A segment was excluded from the analysis, if the ratio of valid data points in the segment was <80%. In the segments analyzed, defected parts of data, if any, were interpolated by the horizontal-step function. After correcting for the loss of variance resulting from the window process, power spectral density was integrated over 0.04–0.15 Hz and 0.15–0.45 Hz for assessing the power of low frequency (LF) and high frequency (HF) components, respectively. The power of these components was converted into mean amplitude ([2 × power]^1/2^) to reduce the skewness from the normal distribution.

### Statistical analysis

The agreement between PRV and HRV measures was evaluated from two aspects; (1) agreement when these measures are used for assessing intra-individual variations and (2) agreement when they are used for inter-individual comparisons.

To examine the former agreement, minute-to-minute pulse rate and heart rate and spectral components of PRV and HRV for 5-min segments were compared within each subject. The agreement between corresponding values were evaluated with (a) the difference-against-mean plot and the 'limits of agreement' of Bland and Altman method [19] and (b) intraclass correlation coefficients for 2-way mixed effects analysis of variance with defining *segments *as the random factor and methods as the fixed factor [20]. The statistical adequacy of the segments as random factor is partly supported by the fact that long-term heart rate fluctuation has the characteristics of random fractal noise [1,21].

To examine the latter agreement for inter-individual comparisons, the minute-to-minute and 5-min segment values were averaged over the entire recording length for individual subjects. The agreement between the corresponding mean values were evaluated with (a) the Bland and Altman method same as above [19] and (b) intraclass correlation coefficients for 2-way mixed effects analysis of variance with defining *subjects *as the random factor and methods as the fixed factor [20].

Data were presented as mean (SD) or median (range). Type 1 error level was set at probability (p value) of < 0.05.

## Results

### Simulation studies

Simulation studies were performed for evaluating the performance of the PFDM, particularly the effects of (1) Fr adaptation with data segmentation, (2) Fr optimization with iterative algorithm, (3) Fc convergence, and (4) robustness against the fluctuation in pulse height and baseline trend. Also, the frequency characteristics of the PFDM were examined to test if the output from the PFDM is appropriate for frequency domain analyses.

### Effects of Fr adaptation and iterative optimizations

**Figure 4:**
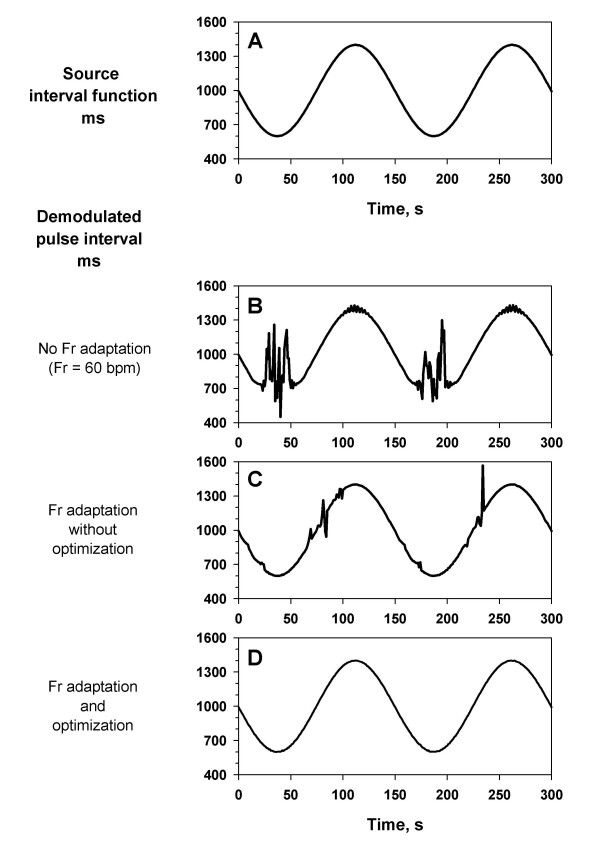
**Effects of adaptation and optimization algorithms. **Simulated pulse wave signal was generated by a sinusoidal source interval function (panel A) that fluctuated between 600 and 1400 ms (corresponding pulse rate, from 43 to 100 bpm). Panel B: the demodulated pulse interval with fixed Fr (60 bpm) and Fc (40 bpm). Panel C: the demodulated pulse interval with data segmentation (length, 30 s with 10-sec overlapping at both end) and Fr adaptation (Fc was set at Fr/3). Panel D: the demodulated pulse interval with data segmentation, Fr adaptation and Fr optimization with iteration (Fc was set at Fr/3).

Simulated pulse wave signal was generated with modulating the pulse interval by a sinusoidal source interval function (Fig. 4A) that fluctuated between 600 and 1400 ms (corresponding to 43 to 100 bpm). The signal was analyzed with PFDM algorithms with different levels of Fr adjustment. Fig. 4B is the result with an algorithm with fixed Fr at 60 bpm and Fc at 40 bpm (no adaptation). Although the range of CDM filter covered pulse rate from 20 to 100 bpm, the output is affected by the 2nd harmonic and the 2nd subharmonic when the pulse interval reached the upper and lower ends. This problem was partly resolved with an algorithm furnishing Fr adaptation with data segmentation (length 30 s with overlapping 10 s at both ends) and an appropriate Fc (Fr/3; Fig. 4C); however, estimation errors still occurred at the portions where the pulse interval changed fast, due to transient deviation of the pulse frequency outside the range of CDM filter (spectral leakage). Finally, the pulse interval was satisfactorily estimated with adding iterative Fr optimization for each segment (Fig. 4D). The estimation error (differences between the estimated pulse intervals and the source interval function) was within ± 2 ms. The median (range) of the number of iteration with a tolerance of 0.001 bpm was 3 (3–5).

### Effects of Fc convergence

**Figure 5:**
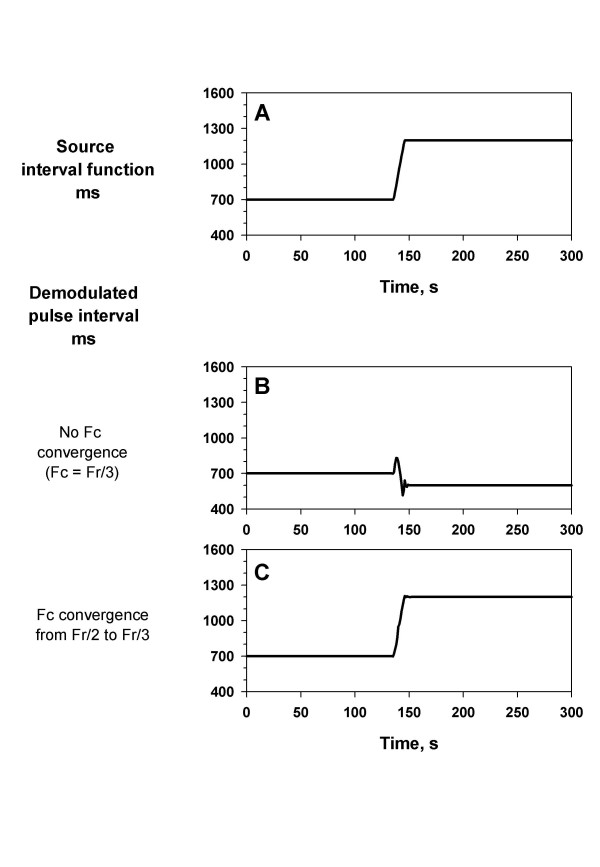
**Effects of convergence algorithm. **Simulated pulse wave signal was generated by a source interval function with an abrupt increase from 700 to 1200 ms during 10 s (panel A). Panel B: demodulated pulse interval with data segmentation, Fr adaptation and Fr optimization (iteration) without Fc convergence (Fc was set at Fr/3). Panel C: demodulated pulse interval with data segmentation, Fr adaptation, Fr optimization (iteration) and Fc convergence (Fc was set at Fr/2 initially and then converged to Fr/3).

The PFDM algorithm even furnishing the Fr adaptation and optimization failed to follow this rapid change due to spectral leakage and erroneous adaptation to the harmonics or subharmonics of pulse wave (Fig. 5B). This possibility can be reduced with widening the range of CDM filter, which subsequently converged to an appropriate width after the fundamental pulse wave is detected. In the PFDM algorithm, the Fc was set at Fr/2 at the first iteration and then converged to Fr/3 in the following iterations, by which the rapid change in pulse interval was detected appropriately (Fig. 5C).

### Robustness against pulse height and baseline fluctuations

**Figure 6:**
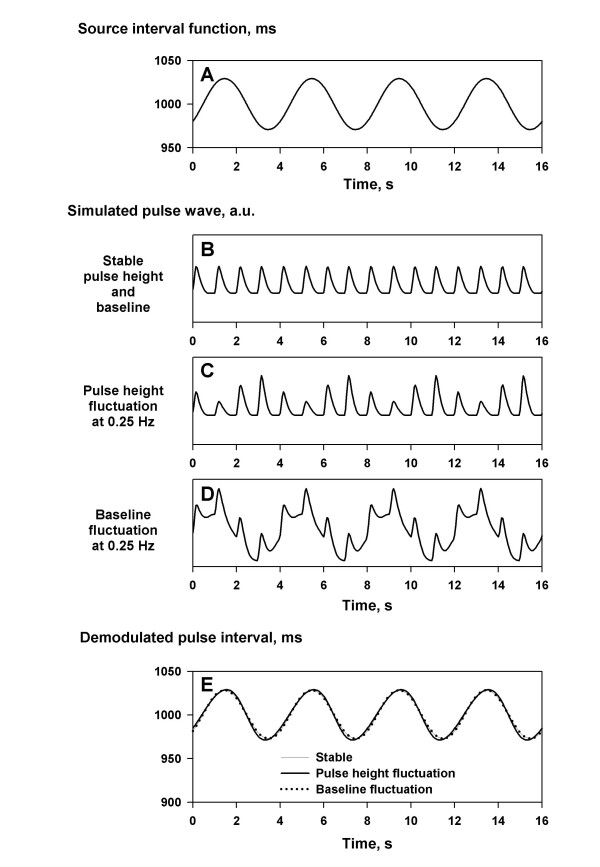
**Effect of pulse height and baseline fluctuations. **Simulated pulse wave signals were generated by a source interval function oscillating at 0.25 Hz (panel A) with stable pulse height and baseline (panel B), with fluctuating pulse height (panel C), and with fluctuating baseline (panel D). These fluctuations had no substantial influence (the estimation error, within ± 2 ms) on the results of the PFDM (panel E).

Fluctuations of pulse height and baseline, such as those due to respiration and body movements, are quite common. Simulation study, however, revealed that such fluctuations have only small influence on the estimation of pulse interval by the PFDM (Fig. 6).

### Frequency characteristics

**Figure 7:**
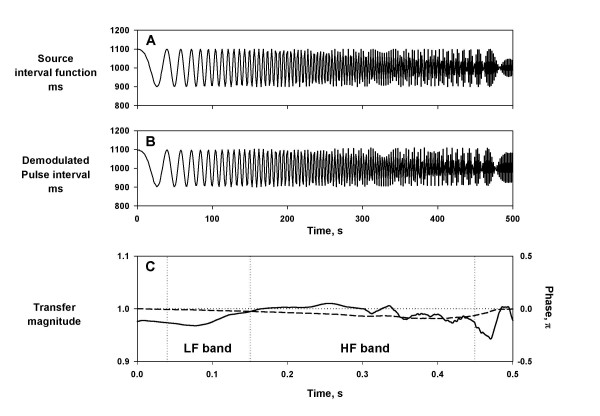
**Frequency characteristics of PFDM. **Simulated pulse wave signal was generated by a source interval function oscillating at linearly increasing frequency form 0 to 0.5 Hz at 0.001 Hz/sec (panel A). Panel B: demodulated pulse interval obtained from the simulated pulse wave with the PFDM. Panel C: transfer magnitude (solid line) and transfer phase (dashed line) calculated between the source interval and the demodulated pulse interval with the PFDM. Insets indicate the frequency ranges corresponding low frequency (LF) and high frequency (HF) bands.

To test the transfer function of PFDM, the simulated pulse wave was generated using an oscillatory source interval function with linearly increasing frequency from 0 to 0.5 Hz at 0.001 Hz/sec (Fig. 7A). The fluctuation of demodulated pulse interval by the PFDM appeared to reflect faithfully both frequency and amplitude between 0 and 0.43 Hz (Fig. 7B). The transfer magnitude and phase between the source and demodulated pulse interval from 0.00 to 0.43 Hz were between 0.97 and 1.02 and between -0.1 and 0 π, respectively (Fig. 7C).

### Analysis of actual data

Actual data of overnight recordings of pulse wave together with simultaneous ECG were obtained from 30 subjects out of 33. Data were lost due to technical problems in a subject and were excluded due to atrial fibrillation during the entire recording periods in two subjects. The mean ± SD length of data in the 30 subjects was 6.0 ± 0.8 hr. In these data, median ratio (range) of 5-min segments that met the criteria (valid data ≥80%) for spectral analysis was 87 (63–95) %.

The presences of atrial fibrillation in the two excluded subjects were detected by the PFDM of pulse wave. In both subjects, more than 90% of the pulse intervals deviated 12% or more from the local moving average and none of the 5-min segments met the inclusion criteria (valid data ≥80%).

### Agreement between pulse rate and heart rate

**Figure 8:**
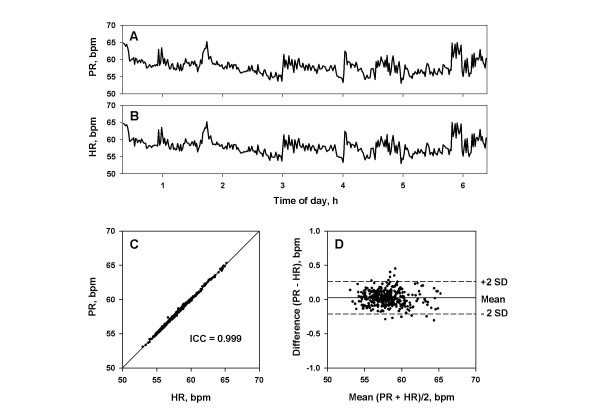
**Agreement of demodulated pulse rate by PFDM and ECG heart rate. **Panels A and B: minute-to-minute pulse rate (PR) demodulated by the PFDM (panel A) and minute-to-minute heart rate (HR) from simultaneously recorded ECG (panel B) in a representative subject during all-night sleep. Panel C: correlation between PR and HR with the intraclass correlation coefficient (ICC). Panel D: the Bland and Altman plot with the upper and lower limits of agreement (+2 SD and -2 SD, respectively). SD = standard deviation.

Minute-to-minute pulse rate assessed by the PFDM showed overnight trends almost identical to those of minute-to-minute heart rate assessed by ECG (Fig. 8). Good agreement was observed between pulse rate and heart rate within each subject (Fig. 8C and 8D). For 30 subjects, the median (range) of intraclass correlation coefficients was 0.999 (0.998–0.999), the mean (SD) of the differences between them was -0.00 (0.03) bpm and the upper and lower limits of agreement were 0.21 (SD, 0.06) and -0.22 (SD, 0.09) bpm, respectively.

The analysis of agreement for inter-individual comparisons showed almost perfect agreement between the mean pulse rate and heart rate of individual subjects. The intraclass correlation coefficient was 0.999, the mean difference was 0.00 bpm, and the upper and lower limits of agreement were 0.03 and -0.03 bpm, respectively.

### Agreement of spectral components of PRV and HRV

**Figure 9:**
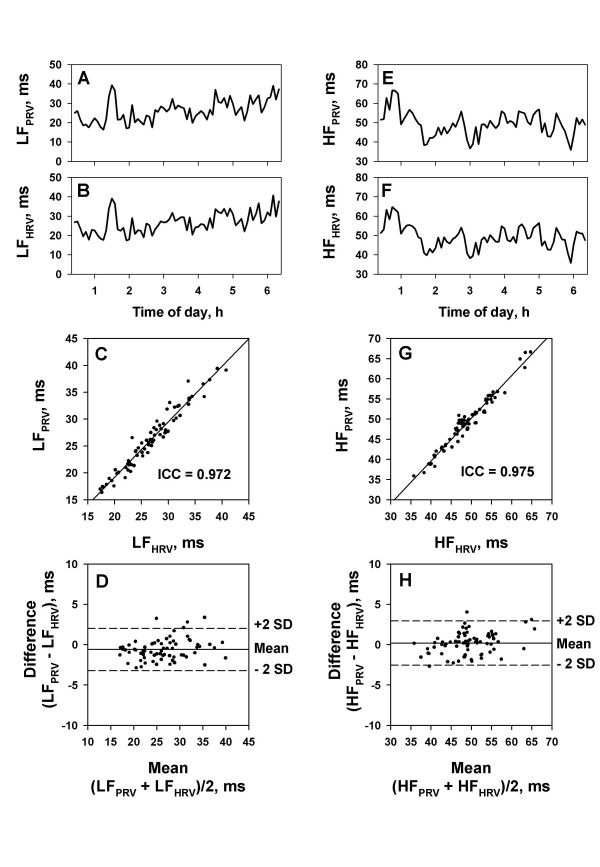
**Agreement of spectral components of pulse rate variability (PRV) and heart rate variability (HRV). **The amplitudes of spectral components were calculated for sequential 5-min segments over the entire length of data in a representative subject. Left panels: trendgram (panels A and B), scatter plot (panel C) and the Bland and Altman plot (panel D) for the amplitudes of low-frequency components of pulse interval variability (LF_PRV_) and R-R interval (LF_HRV_). Right panels: trendgram, scatter plot and the Bland and Altman plot of the amplitudes of high-frequency components of pulse interval variability (HF_PRV_) and R-R interval (HF_HRV_). ICC = intraclass correlation coefficient. Broken lines in the Bland and Altman plots represent the upper and lower limits of agreement (+2 SD and -2 SD, respectively). SD = standard deviation.

The amplitude of LF and HF components of PRV and HRV were calculated for sequential 5-min segments of demodulated pulse interval by PFDM and ECG R-R interval, respectively. The overnight trends of amplitude of both components were similar between PRV and HRV (Fig. 9). The analysis of agreement between them showed unexpectedly good agreements (Fig. 9). For 30 subjects, the median (range) of intraclass correlation coefficients for the LF and HF amplitudes were 0.989 (0.911–0.998) and 0.940 (0.676–0.990), respectively. The absolute means (SD) of the differences were -0.1 (0.6) and 0.1 (0.5) ms for both LF and HF amplitudes and the upper and lower limits of agreement were 2.1 (SD, 1.3) and -2.4 (SD, 1.3) ms for LF amplitude and 4.1 (SD, 2.2) and -3.9 (SD, 1.6) ms for HF amplitude.

**Table 1:** Agreement between PRV and HRV for inter-individual comparisons

	N	PRV Mean (SD) ms	HRV Mean (SD) ms	Mean difference ms	Upper limit of agreement ms	Lower limit of agreement ms	ICC
LF	30	27.4 (7.5)	27.6 (7.3)	0.2	1.2	-0.7	0.997
HF	30	31.6 (10.7)	31.4 (10.5)	-0.2	3.9	-4.4	0.981

Analyses were performed for mean values of individual subjects.

HF = high frequency component, HRV = heart rate variability, ICC = intraclass correlation coefficient, LF = low frequency component, PRV = pulse rate variability, SD = standard deviation.

To evaluate the agreement for inter-individual comparisons, mean values of LF and HF amplitude of individual subjects were compared between PRV and HRV. The results showed good agreement for the mean LF amplitude and acceptable agreement for the mean HF amplitude (Table 1).

### Influence of ventricular ectopies (VE)

In this study, *abnormal *pulse intervals caused by ectopies and heart blocks were excluded whenever they deviated 12% or more from the local average. To examine the influence of this process on the assessments of pulse rate and spectral components, the effects of the frequency of VE on the agreement between values calculated from the pulse interval by PFDM and ECG R-R interval were analyzed.

**Figure 10:**
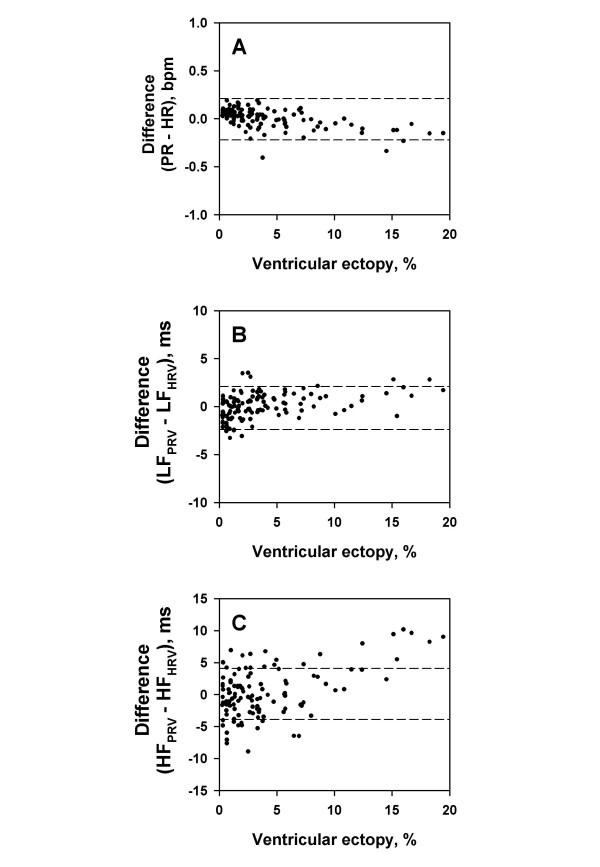
**Influence of ventricular ectopies on assessment by PFDM. **The amplitudes of spectral components were calculated for 5-min sequential segments of demodulated pulse interval by PFDM and ECG R-R interval in three subjects with frequent ventricular ectopies. Panel A: the difference in pulse rate (PR) and heart rate (HR). Panel B: the difference in the amplitude of low-frequency component of pulse interval (LF_PRV_) and that of R-R interval (LF_HRV_). Panel C: the difference in the amplitude of high-frequency component of pulse interval (HF_PRV_) and that of R-R interval (HF_HRV_). All data are plotted against percentage of ventricular ectopies within each 5-min segment.

Of the 30 subjects, only 3 subjects had relatively frequent VE (187, 676, and 699 beats, respectively, during the overnight recordings). In these 3 subjects, when the frequency of VE in 5-min segments was <~10%, VE showed no substantial effects on the agreement between the values calculated from two methods (Fig. 10). When the frequency of VE was >~10%, however, the HF amplitude of PRV was greater than that of HRV (Fig. 10C).

## Discussion

In this study we proposed the PFDM technique as a method for demodulating instantaneous pulse rate from pulse wave signal and demonstrated its usefulness for assessing spectral components of PRV. The simulation studies revealed that (1) the PFDM provides reliable measurement of instantaneous pulse rate even if it fluctuates rapidly and over a wide range, (2) it is robust to the variations of pulse height and baseline trend, and (3) it preserves the frequency characteristics of the source modulator function, a feature necessary for spectral analysis. The analysis of actual pulse wave revealed that (1) minute-to-minute pulse rate assessed by the PFDM agreed perfectly with minute-to-minute heart rate measured by ECG, (2) the amplitudes of LF and HF components of PRV show overnight trends quite similar to those of HRV, and (3) the mean values of LF and HF amplitudes during night show good agreement between PRV and HRV. These observations indicate that the PFDM provides a reliable assessment of pulse rate and PRV and suggest that this technology makes pulse wave a potentially useful source signal for assessing cardiovascular variability and dynamics.

It is noteworthy that the pulse wave was sampled at a frequency of 20 Hz, while the ECG was sampled at 125 Hz. Nevertheless, we observed surprisingly good agreement not only between pulse rate and heart rate but also between the spectral components of PRV and HRV. This indicates that the accuracy of the PFDM analysis of pulse interval is not directly dependent on the time resolution of data. Indeed, the simulation studies revealed that the estimation errors of pulse interval by the PFDM are within ± 2 ms despite the fact that the sampling interval is 50 ms (20 Hz). This seems attributable to the fact that only PFDM, but not R-R interval measurement, utilizes the periodicity of signals; i.e., the PFDM analyzed pulse wave with assuming it as a cosine function with slowly changing amplitude and phase. Even for ECG, a periodicity analysis method can estimate instantaneous heart rate from signals sampled at a low frequency (5 Hz) [22].

Although measurement of R-R intervals of ECG is the standard for HRV analysis, this method has practical limitations [1]. Recordings of ECG require attachment of multiple electrodes and cables, which limit applications of HRV analysis in public or home health cares. Also, R-R intervals are intervals of event series. An appropriate interpolation is necessary to estimate the underlying modulator function that is the putative subject of HRV analysis. For this purpose, several interpolation methods with differing mathematic features have been proposed; but convincing physiological reasoning for selecting a method is lacking.

In contrast to ECG, recording of pulse wave requires only a single sensor, which allowed development of devices that can be used in daily life even everyday. Also, smooth waveform of pulse wave requires a lower sampling frequency (10–20 Hz) and its sinusoidal feature is advantageous to the PFDM as mentioned above. Furthermore, by the use of the PFDM, the signal putatively modulating the pulse interval is directly extracted as a continuous function. The signal can be used directly for spectral analysis without interpolation processes.

An apparent limitation of the PFDM of pulse wave is inability to assess the type of arrhythmias. A substantial part of such arrhythmia, however, can be detected by using appropriate criteria such as those using deviation of intervals from the local mean. In fact, the PFDM was able to detect persistent atrial fibrillation in this study. Also, even for the segments including frequent VE, the agreement for the pulse rate and LF amplitude are unaffected, although the HF amplitude was *overestimated *for the segments including VE>~10% compared with the HF amplitude obtained by R-R interval analysis. Interestingly, however, the exclusion of arrhythmic data has been reported to result in an *underestimation *of the HF amplitude for HRV analysis with R-R interval [18]. It should be also noted that the optimal criteria for excluding abnormal beats are not uniform but subject to change depending on age and other conditions that could affect the magnitude of physiological PRV. This issue, however, is not specific to PFDM but common to PRV and HRV assessment at least in detection of atrial ectopic beats and heart blocks.

Another limitation of the PFDM may be caused by the physiological differences between pulse interval and R-R interval. Theoretically, the variability of pulse rate is the sum of the variability existing in R-R interval, pre-ejection period and pulse wave velocity. Constant et al [14] suggested that, in the standing position, respiratory fluctuation of pulse wave velocity might be important cause of respiratory pulse rate variation. Although the present study indicates usefulness of the PFDM for assessing PRV and good agreement between PRV and HRV during night sleep, its usefulness as a surrogate of HRV for assessing autonomic functions and mortality risk need to be examined *de novo*.

## Conclusion

The PFDM of pulse wave signal provides a reliable assessment of PRV. Given the popularity of pulse wave equipments, this technology may open new ways to studies of long-term assessment of cardiovascular variability and dynamics among general populations.

## Authors' contributions

JH conceived and designed the study, collected the data and drafted manuscript. AKB participated in the conception of the study and revised the manuscript critically. AK participated in the design of the study and was involved in acquisition of data and revising the manuscript critically. NO was involved in analysis of data and revising the manuscript. FY participated in the conception of the study and was involved in acquisition of data and revising the manuscript. All authors read and approved the final manuscript.
